# Supplementary material for: Nano-Silver-Loaded Activated Carbon Material Derived from Waste Rice Noodles: Adsorption and Antibacterial Performance
Source: Nanomaterials (Basel). 2024 Nov 20;14(22):1857. doi: 10.3390/nano14221857 (PMC11597512; doi:10.3390/nano14221857)
Supplement: Supplementary file 1 [file nanomaterials-14-01857-s001.zip › nanomaterials-3287756-supplementary.pdf]

nanomaterials

Supplementary Information

Nano-Silver-Loaded Activated Carbon Material Derived from Waste Rice Noodles: Adsorption and Antibacterial Performance

Guanzhi Ding, Guangzhi Qin, Wanying Ying, Pengyu Wang, Yang Yang, Chuanyang Tang, Qing Liu, Minghui Li, Ke Huang and Shuoping Chen *

College of Materials Science and Engineering, Guilin University of Technology, Guilin 541004, China; 1020220195@glut.edu.cn (G.D.); 1020230210@glut.edu.cn (G.Q.); 2120210327@glut.edu.cn (W.Y.); 2120220368@glut.edu.cn (P.W.); 1020230205@glut.edu.cn (Y.Y.); 2120230405@glut.edu.cn (C.T.); 2120220336@glut.edu.cn (Q.L.); 2120230354@glut.edu.cn (M.L.); 3222042041416@glut.edu.cn (K.H.)

* Correspondence: 2012014@glut.edu.cn

S1. General characterization

The characterization of the Ag/AC composite material was performed with reference to similar characterization methods as outlined in our prior reports[27-30]. The powder X-ray diffraction (PXRD) patterns of the Ag/AC composite and other control samples were acquired via an X'pert PRO X-ray diffractometer (Panalytical, Malvern, Worcestershire, UK) employing Cu Kα radiation (λ = 0.15418 Å) at 40 kV and 40 mA, with the scanning speed set at 5° per minute (2θ). The scanning electron microscope (SEM) images and energy-dispersive X-ray (EDX) spectra of the Ag/AC were obtained by using an S-4800 field emission scanning electron microscope (Hitachi, Chiyoda, Tokyo, Japan) with an accelerating voltage of 20 kV. The transmission electron microscope (TEM) images were obtained using a JEM-2100F field emission transmission electron microscope from JEOL (Akishima, Tokyo, Japan) at an accelerating voltage of 200 kV. The obtained infrared (IR) spectra of the resultant products were recorded as KBr pellets in the range of 400 to 4000 cm^{-1}, utilizing a Nicolet 5700 FT-IR spectrometer (Thermo Fisher, Waltham, MA, USA) featuring a spectral resolution of 4.00 cm^{-1}. X-ray photoelectron spectroscopy (XPS) for both the Ag/AC composite and AC was executed using an ESCALAB 250Xi X-ray photoelectron spectrometer (Thermo Fisher, Waltham, MA, USA), powered by an Al Kα X-ray as the excitation source. The determination of BET-specific surface areas for both the Ag/AC composite and AC was executed using a TriStar II 3020 surface area analyzer (Micromeritics, Atlanta, GA, USA) in a nitrogen environment. The assessment was conducted with an initial temperature of 150 °C and a heating rate of 10 °C per second. To ascertain the points of zero charge (PZC) for the obtained samples, the following method was employed[27,28]: A sealed centrifugal tube containing 10 mL of 0.01 mol L^{-1} NaCl solution was employed. The pH of the solution was manipulated to fall between 2 and 11 through incremental additions of HCl or NaOH solutions. Subsequently, 0.05 g of the sample was introduced, and the final pH was gauged after 10 hours of agitation. The PZC was determined at the intersection of the pH final versus pH initial curve for the test sample and the control sample.

S2. Measurement of adsorption performance of Ag/AC composite

Similar to the method employed in our previous work[27], Cr(VI) was used to test the adsorption performance of the obtained Ag/AC composite. Before the experiment, the Cr(VI) was prepared as aqueous solutions with $K_2Cr_2O_7$ as the element source. Then, the Cr(VI) solution was mixed with the Ag/AC composite with an adsorbent dosage of 1 g·L^{-1} in a crystallizing dish with continuous magnetic stirring. After absorption, the resulting solution was centrifugated, and the concentration of Cr(VI) was tested with diphenylcarbazide spectrophotometry. The adsorption capacity of AC to Cr(VI) was calculated according to the spectrophotometry results using the following equation:

$$q_e = \frac{(C_0 - C_e)}{AD} \qquad \text{(S1)}$$

Herein, q_e is the equilibrium adsorption capacity of the adsorbent in mmol·g^{-1}; C_0 is the initial concentration of the heavy metal ions in mg·L^{-1}; C_e is the equilibrium concentration of the heavy metal ions after adsorption in mg·L^{-1}; A is the atomic weight of heavy metal in g/mol; and D is the adsorbent dosage in g·L^{-1}.

The adsorption equilibrium was established within 12 h for both instance of Cr(VI) adsorption, with the initial concentration of heavy metal element ranging from 50–400 mg·L^{-1}.

The Langmuir and Freundlich adsorption isotherms were applied to describe the adsorption of AC to Cr(VI) or Pb(II) at room temperature.[48] The Langmuir equations are as follows:

$$q_e = \frac{q_m b C_e}{1 + bC_e} \text{ (nonlinear form) or } \frac{C_e}{q_e} = \frac{1}{bq_m} + \frac{1}{q_m} C_e \text{ (linear form)} \quad (S2)$$

where q_m is the maximum amount of heavy metal uptake in mmol·g^{-1}; and b is the constant that refers to the bonding energy of adsorption related to free energy and net enthalpy in L·mg^{-1}.

The Freundlich isotherm is given as follows:

$$q_e = k_f C_e^{1/n} \text{ (nonlinear form) or } \log q_e = \log k_f + \frac{1}{n} \log C_e \text{ (linear form)} \quad (S3)$$

where k_f is the constant related to the adsorption capacity of the adsorbent in mmol·g^{-1}, and $1/n$ is the intensity of the adsorption constant.

The temperature-dependent experimental data for the uptake of Cr (VI) by the synthesized Ag/AC composite were analyzed to determine key thermodynamic parameters, including Gibbs free energy (ΔG), enthalpy (ΔH), and entropy (ΔS).[49] The standard Gibbs free energy change (ΔG) is expressed through Equation 4:

$$\Delta G = -RT \ln K_{eq} \quad (S4)$$

Here, T represents the absolute temperature in Kelvin (K), R is the gas constant (8.314 J·mol^{-1}·K^{-1}), and K_{eq} is the equilibrium constant, which relates the concentrations of the adsorbed and bulk phases, defined as follows (Equation 5):

$$K_{eq} = \frac{C_{ads}}{C_e} = \frac{(C_0 - C_e)}{C_e} \quad (S5)$$

In this equation, C_{ads} and C_e correspond to the concentrations of Cr (VI) on the adsorbent and in the aqueous solution (mg·L^{-1}), respectively. The changes in enthalpy (ΔH, kJ·mol^{-1}) and entropy (ΔS, kJ·mol^{-1}·K^{-1}) are connected to the Gibbs free energy (ΔG, kJ·mol^{-1}) through Equation 6:

$$\Delta G = \Delta H - T\Delta S \quad (S6)$$

By substituting Equation 4 into Equation 6, Equation 7 is obtained.

$$\ln K_{eq} = -\frac{\Delta H}{RT} + \frac{\Delta S}{R} \quad (S7)$$

The values of ΔH and ΔS were derived from the slope and intercept of the linear plot of ln K_{eq} against 1/T.

The adsorption dynamics was tested with an initial Cr(VI) concentration of 150 mg/L with a different adsorption time ranging from 10 to 120 min, and the results were fitted by pseudo-first-order or pseudo-second-order mode.[50] The pseudo-first-order equation used was as follows:

$$\log(q_e - q_t) = \log q_e - \frac{K_1}{2.303} t \quad (S8)$$

where q_e and q_t are the heavy metal uptake at equilibrium and time t, respectively, and K_1 is the constant of first-order adsorption in min^{-1}.

The pseudo-second-order equation is given as follows:

$$\frac{t}{q_t} = \frac{1}{K_2 q_e^2} + \frac{t}{q_e} \tag{S9}$$

where K_2 is the rate constant of second-order adsorption in g mmol^{-1} min^{-1}.

The whole regression analysis was carried out in OriginPro software (OriginPro 2019, Originlab, Northampton, MA, USA) in order to predict the parameters.

The procedure to assess the removal efficiency of Cr(VI) by the obtained Ag/AC composite in different water types, including deionized water, tap water, and river water (collected from the Li River in Guilin, China), was conducted as follows: a Cr(VI) source was dissolved in each type of water to prepare solutions with an initial Cr(VI) concentration of 100 mg/L, and the Ag/AC composite was added at a dosage of 1 g·L^{-1}. Samples were taken at regular adsorption intervals to analyze the Cr(VI) removal efficiency across the three water types (ultrapure water, lake water, and tap water). The Cr(VI) removal efficiency was determined by evaluating the C/C_0 value, where C represents the Cr(VI) concentration at a specific time, and C_0 symbolizes the initial Cr(VI) concentration. Each data point represents the average measurement from three identical samples.

The regeneration and reusability experiments of the Ag/AC composite followed these steps: Initially, the Ag/AC composite was used to adsorb Cr(VI) from a solution with an initial concentration of 100 mg/L at a dosage of 1 g·L^{-1} over a 120-minute period. The Cr(VI)-adsorbed Ag/AC composite was then mixed with deionized water at a ratio of 1:100, and the pH of the mixture was adjusted to 12 by adding a 1 mol/L NaOH solution. The mixture was shaken at room temperature for 24 hours to achieve complete desorption of Cr(VI). After shaking, the mixture was filtered, and the Ag/AC composite was washed to neutrality with deionized water, then oven-dried at 80 ℃, completing the regeneration process. The regenerated Ag/AC composite was then reused for Cr(VI) adsorption from a 100 mg/L Cr(VI) solution, with six adsorption–regeneration cycles in total. The Cr(VI) removal efficiency was again determined by evaluating the C/C_0 value.

S3. Measurement of antibacterial performance of Ag/AC composite.

The antibacterial performance test was conducted using *E. coli* and *S. aureus* as target bacterial strains. Taking *E. coli* as an example, 25 g of LB solid medium powder was dissolved in 1 L of water, and the solution was sterilized using high-temperature and high-pressure methods to obtain the LB medium. Subsequently, 60 mg of the Ag/AC composite and AC (control sample) were added to 50 mL centrifuge tubes, which were sterilized under UV light for 90 minutes. In a biosafety cabinet, 30 mL of the sterilized LB medium was added to each tube, and the mixtures were shaken thoroughly for later use. Meanwhile, the cultured *E. coli* was diluted with sterile LB to prepare the bacterial suspension. A mixture of 1 mL *E. coli* suspension and 1 mL LB medium was tested for absorbance at 600 nm and 37°C using a microplate reader. Next, 1 mL of *E. coli* suspension and 1 mL of LB medium containing the Ag/AC or AC sample were mixed in 15 mL centrifuge tubes, shaken, and placed in a shaker at 37 °C with a speed of 200 rpm for 1 hour. After removing the tubes and allowing them to stand for 5 minutes, samples were taken, and the absorbance of E. coli was measured at 600 nm and 37°C using a microplate reader. The procedure was repeated for 2, 3, and 4 hours of shaking, with absorbance measurements taken at each interval. The antibacterial rate of the Ag/AC composite and the AC control sample at different contact times was calculated based on the absorbance data. The antibacterial rate of *S. aureus* was tested using the same method.

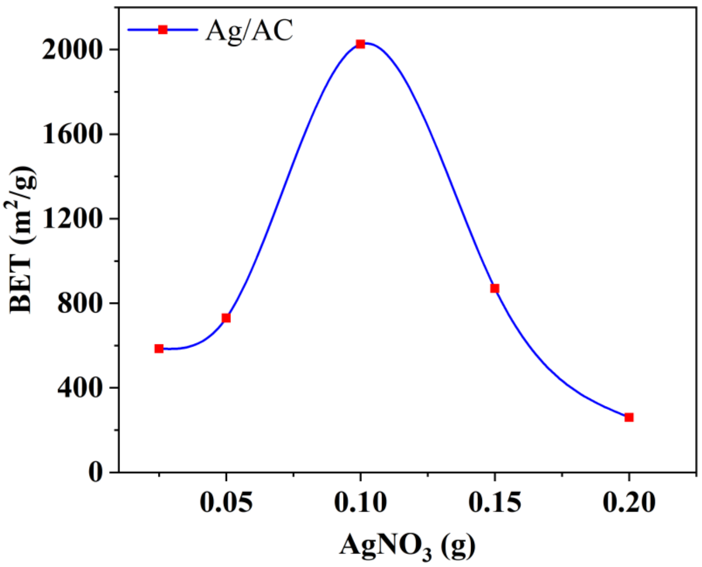

Figure S1. The relationship between the $AgNO_3$ dosage and the specific surface area of the resulting Ag/AC material, with a constant HTC input of 2g for all samples. The other preparation conditions included a mass ratio of HTC to NaOH of 1:3, a calcination temperature of 900 °C, and a calcination time of 1 hour. It can be observed that the maximum specific surface area of the Ag/AC material was achieved when the mass ratio of $AgNO_3$ to HTC was 1:20.

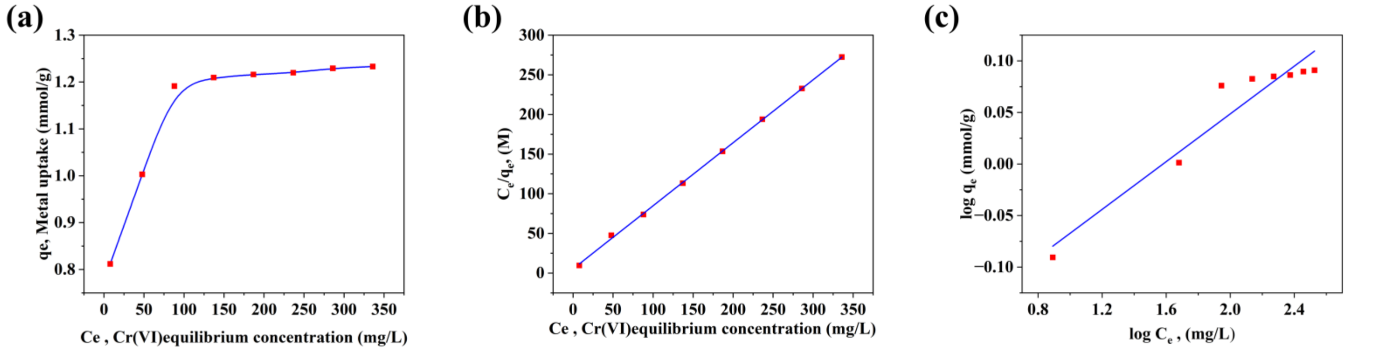

Figure S2. The adsorption isotherm of AC without Ag loading to Cr(VI) using the equilibrium concentration (a), Langmuir equation (linear form, (b)), and Freundlich equation (linear form, (c))

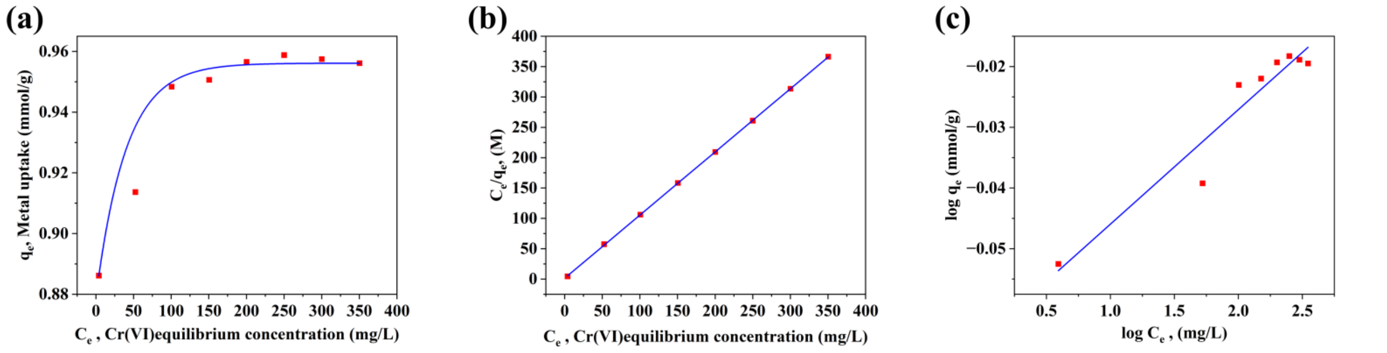

Figure S3. The adsorption isotherm of commercial coal-based AC to Cr(VI) using the equilibrium concentration (a), Langmuir equation (linear form, (b)), and Freundlich equation (linear form, (c))

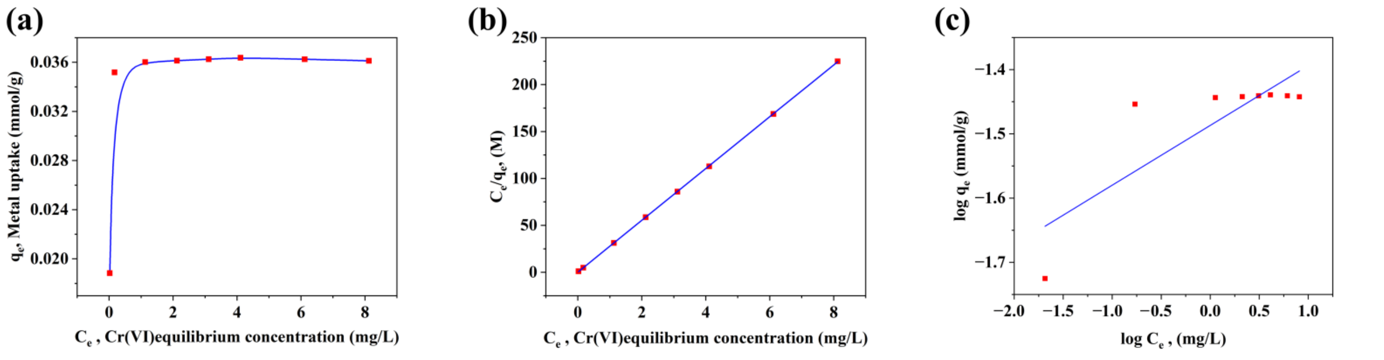

Figure S4. The adsorption isotherm of WRN-based HTC to Cr(VI) using the equilibrium concentration (a), Langmuir equation (linear form, (b)), and Freundlich equation (linear form, (c))

Table S1. The optimization of the experimental conditions of the Ag/AC composite.

Serial Number	Mass Ratio of HTC to NaOH	Activation Temperature (°C)	Activation Time (hour)	Surface Area (m^2/g)
1#	1:1	700	0.5	214.59
2#	1:1	800	1	43.71
3#	1:1	900	1.5	355.37
4#	1:2	700	1	845.52
5#	1:2	800	1.5	93.79
6#	1:2	900	0.5	128.64
7#	1:3	700	1.5	724.86
8#	1:3	800	0.5	1234.49
9#	1:3	900	1	2025.96
Average value for leve1 1	204.56	594.99	525.91	
Average value for leve1 2	355.98	457.33	971.73	
Average value for leve1 3	1328.44	836.66	391.34	
Range	1123.88	379.33	580.39	

In this study, while maintaining a mass ratio of $AgNO_3$ to HTC at 1:20, a three-factor, three-level orthogonal experiment was conducted to optimize the mass ratio of HTC to NaOH, activation temperature, and activation time. Based on the results of the specific surface area tests, the optimized synthesis parameters obtained were a mass ratio of HTC to NaOH of 1:3, a calcination temperature of 900 °C, and a calcination time of 1 hour, which corresponded to the parameters designed for sample 9. The Ag/AC composite material prepared using this optimized process exhibited a high specific surface area of 2025.96 m^2/g.

Table S2. The adsorption parameters of the Langmuir and Freundlich isotherms of AC without Ag loading for the adsorption of Cr(VI) at room temperature.

Langmuir models			Freundlich models		
q_m (mmol·g^{-1})	b (L·mg^{-1})	R^2	k_f (mmol·g^{-1})	1/n	R^2
1.2584	0.146	0.9996	0.6562	0.116	0.9245

Table S3. The adsorption parameters of the Langmuir and Freundlich isotherms of commercial coal-based AC for the adsorption of Cr(VI) at room temperature.

Langmuir models			Freundlich models		
q_m (mmol·g^{-1})	b (L·mg^{-1})	R^2	k_f (mmol·g^{-1})	1/n	R^2
0.9612	0.745	0.9999	0.8614	0.0189	0.9259

Table S4. The adsorption parameters of the Langmuir and Freundlich isotherms of WRN-based HTC for the adsorption of Cr(VI) at room temperature.

**Langmuir models**			**Freundlich models**		
q_m **(mmol·g⁻¹)**	**b (L·mg⁻¹)**	R^2	k_f **(mmol·g⁻¹)**	**1/n**	R^2
0.036	215.04	0.9999	0.0326	0.0931	0.6888

References

27. Jin, X.Y.; Che, R.J.; Yang, J.; Liu, Y.; Chen, X.B.; Jiang, Y.E.; Liang, J.Q.; Chen, S.P.; Su, H.P. Activated carbon and carbon quantum dots/titanium dioxide composite based on waste rice noodles: simultaneous synthesis and application in water pollution control. *nanomaterials* **2022**, *12(3)*, 472.
28. Jin, X.Y.; Ying, W.Y.; Che, R.J.; Xiao, P.; Zhou, Y.Q.; Liu, Y.; Liu, M.Y.; Chen, S.P. CQDs/ZnO composites based on waste rice noodles: preparation and photocatalytic capability†. *RSC Adv.* **2022**, *12(36)*, 23692-23703.
29. Che, R.J.; Tu, B.Y.; Zhu, Y.N.; Miao, J.H.; Jin, X.Y.; Chen, S.P. Waste rice noodle-based CQDs/ZnO composite nanorod array on steel wire mesh: Preparation and photocatalytic capability. *Arabian J. Chem*. **2023**, *16(11)*, 105231.
30. Ying, W.Y.; Liu, Q.; Jin, X.Y.; Ding, G.Z.; Liu, M.Y.; Wang, P.Y.; Chen, S.P. Magnetic carbon quantum dots/iron oxide composite based on waste rice noodle and iron oxide scale: preparation and photocatalytic capability. *Nanomaterials*. **2023**, *13(18)*, 2506.
48. Shin, K.Y.; Hong, J.Y.; Jang, J. Heavy metal ion adsorption behavior in nitrogen-doped magnetic carbon nanoparticles: Isotherms and kinetic study. *J. Hazard. Mater.* **2011**, *190*, 36–44.
49. Gorzin, F.; Ghoreyshi, A.A. Synthesis of a new low-cost activated carbon from activated sludge for the removal of Cr (VI) from aqueous solution: Equilibrium, kinetics, thermodynamics and desorption studies. *Korean J. Chem. Eng.* **2013**, *30*, 1594–1602.
50. McKay, G.; Bino, M.J.; Altamemi, A.R. The adsorption of various pollutants from aqueous solutions on to activated carbon. *Water Res.* **1985**, *19*, 491–495.
